# Supplementary material for: Decomposing the Apoptosis Pathway Into Biologically Interpretable Principal Components
Source: Cancer Inform. 2018 May 9;17:1176935118771082. doi: 10.1177/1176935118771082 (PMC5987987; doi:10.1177/1176935118771082)
Supplement: uomf_march_5_2018_pcda_supplement – Supplemental material for Decomposing the Apoptosis Pathway Into Biologically Interpretable Principal Components [file uomf_march_5_2018_pcda_supplement.pdf]

APPENDIX: SUPPLEMENTARY MATERIAL

List of Figures

1 Screeplot of the data and the Broken-Stick model. 5
2 Auer-Gervini step function relating the prior hyperparameter θ to the maximum posterior estimate of the number K of significant principal components. 6
3 Histogram of the estimated dimension for 19 covariance and correlation structures from different methods. Within each plot, from left to right, we show (1) Broken-Stick, (2–3) randomization based procedures, (4) Minka's Laplace approximation, (5) generalized cross-validation, (6–8) PESEL criteria, and (9–16) the Auer-Gervini model with different criteria. Horizontal dotted lines correspond to the true number of PCs over 1000 samples (n=24, m=100). 13
3 (Continued) Histogram of the estimated dimension for 19 covariance and correlation structures from different methods. Within each plot, from left to right, we show (1) Broken-Stick, (2–3) randomization based procedures, (4) Minka's Laplace approximation, (5) generalized cross-validation, (6–8) PESEL criteria, and (9–16) the Auer-Gervini model with different criteria. Horizontal dotted lines correspond to the true number of PCs over 1000 samples (n=24, m=100). 14

List of Tables

1 Values of the absolute difference between the sample test estimates and the known dimension across the correlation matrices with data sets of different number of blocks (1X, 2X) and size (24×100), (96×100), (24×400) and (96×400). Bold values indicate the best results for column settings. 7
2 Percentage of deviations between the estimate from randomization-based procedure (rnd-Lambda) and known dimension. 7
3 Percentage of deviations between the estimate from randomization-based procedure (rnd-Lambda) and known dimension. 8
4 Percentage of deviations between the estimate from the "CPT" criterion in the Auer-Gervini model and known dimension. 8
5 Percentage of deviations between the estimate from the "CPT" criterion in the Auer-Gervini model and known dimension. 9
6 Percentage of deviations between the estimate from "TwiceMean" criterion in the Auer-Gervin model and known dimension. 9
7 Percentage of deviations between the estimate from the Broken-Stick model and known dimension. 10
8 Values of the absolute difference between the sample test estimates and the known dimension across the correlation matrices with data sets of different scenarios under noise level $\sigma_e^2 = 0.01$. Bold values indicate the best results for column settings. 11

9 Values of the absolute difference between the sample test estimates and the known dimension across the correlation matrices with data sets of different scenarios under noise level $\sigma_e^2 = 0.1$. Bold values indicate the best results for column settings. 11
10 Values of the absolute difference between the sample test estimates and the known dimension across the correlation matrices with data sets of different scenarios under noise level $\sigma_e^2 = 1$. Bold values indicate the best results for column settings. 12
11 Number of principal components (PCs) from different algorithms on RPPA data 12

S1. Implementation in PCDimension

In this part, we describe how to compute the significant number of PCs using the R package **PCDimension**, the latest version of which is always available from the R-Forge webpage (`http://r-forge.r-project.org/R/?group_id=1900`). Binary versions of the package can also be installed from the OOMPA R repository using the commands:

```
source("http://silicovore.com/OOMPA/oompaLite.R")
oompainstall("PCDimension")
```

We illustrate the methods by exploring a small simulated data set. First, we load all of the R packages that we need for this analysis. Note that **PCDimension** implements the Broken-Stick model, the randomization-based procedure, and the Auer-Gervini model, while **nFactors** (developed by[18]) is used to run Bartlett's test and its variants.

```
library(PCDimension)
library(nFactors) # implements Bartlett's test
```

Next, we simulate an unstructured data set with random noise. That is, the variation of the data is isotropic and the number of significant PCs is 0. The data is generated via the command:

```
set.seed(12345)
NC <- 200
NS <- 15
ranData <- matrix(rnorm(NS*NC, 6), ncol = NC)
```

Bartlett's Test: Now, we apply Bartlett's test to the simulated data. The required input includes the raw data and the number of attributes (columns).

```
nBartlett(data.frame(t(ranData)), ncol(ranData))

## bartlett anderson   lawley
##       15       15        0
```

The original version of Bartlett's test, and the Anderson variant, fail to return the correct number of components. The Lawley variant does yield the correct value, 0.

Randomization-Based Methods: The **PCDimension** package implements both of the randomization-based statistics that were identified as successful in a previous study [14]. The number of permutations (default, $B = 1000$) and the significance level (default, $\alpha = 0.05$) are optional input arguments in addition to the required data set. The estimated number of PCs is the last point at which the p-value of the statistic of interest greater than the observed one is at least the threshold significance level.

```
rndLambdaF(t(ranData)) # input argument is data

## rndLambda      rndF
##         0         0
```

The randomization-based procedure successfully recovers the true number of PCs.

Broken-Stick: The **PCDimension** package also implements the Broken-Stick model. Both this model and the Auer-Gervini model require the eigenvalues from the singular value decomposition of the data matrix used to compute the principal components. We compute the decomposition using the `SamplePCA` function from the **ClassDiscovery** package, and then extract the variances.

```
spca <- SamplePCA(ranData)
lambda <- spca@variances[1:(NS-1)]
bsDimension(lambda)

## [1] 0
```

In the Broken-Stick model, the individual percentages of variance of the components are compared with the values expected from the "broken stick" distribution. The two distributions are compared element-by-element, and first value $d + 1$ where the expected value is larger than the observed value determines the dimension. The Broken-Stick model also correctly finds that there are zero significant PCs.

Note: In our implementation of the `bsDimension` function, we add an extra parameter (`FUZZ`, with default value 0.005) for this comparison to deal with numerical errors in the estimates of the eigenvalues.

Auer-Gervini: We now use the `SamplePCA` object to construct an Auer-Gervini object.

```
ag.obj <- AuerGervini(spca)
agDimension(ag.obj)

## [1] 0
```

The `agDimension` function takes an optional argument, `agfun` that specifies the method used to automate the computation of the number of PCs. The default value uses the `TwiceMean` method, which correctly concludes that there are zero significant PCs. We can also compare the results of multiple algorithms to automate the procedure.

```
f <- makeAgCpmFun("Exponential")
agfuns <- list(twice = agDimTwiceMean,
               specc = agDimSpectral,
               km = agDimKmeans, km3 = agDimKmeans3,
               tt = agDimTtest,  tt2 = agDimTtest2,
               cpt = agDimCPT, cpm = f)

# compare the list of all criteria
compareAgDimMethods(ag.obj, agfuns)

## twice specc    km   km3    tt   tt2   cpt   cpm
##     0     0     0     0     1     0     0     0
```

Overall, the Auer-Gervini model does an excellent job in selecting the actual number of components since 7 criteria out of 8 return 0 while only the “Ttest” procedure yields 1 which is close enough to 0. If the majority rule is applied, that is, the estimated number of PCs is the one selected in more than 4 criteria in Auer-Gervini model, then we will definitely have 0 as the estimated number of components.

To get a more comprehensive understanding of the Broken-Stick method and the Auer-Gervini model, we use the command to generate the plots of these two models in Figures 1 and 2:

```
bs <- brokenStick(1:NS, NS)
bs0 <- brokenStick(1:(NS - 1), (NS - 1))
pts <- screeplot(spca, ylim = c(0, 0.2))
lines(pts, bs, type = 'b', col = 'blue',
      lwd = 2, pch = 16)
lines(pts[-NS], bs0, type = 'b', col = 'red',
      lwd = 2, pch = 16)
plot(ag.obj, agfuns)
```

Figure 1 shows the broken stick distributions and the relative proportions of the variation that are explained by all the components in the simulated data set. The blue dotted line represents the broken stick distributions under the condition that n equals the number of objects, while the red one means the broken stick distributions after removing the 0 eigenvalue with n equating with the number of objects minus one. And there is almost no difference after removing the effect of eigenvalue 0. The grey rectangles are the relative proportions of the variation explained by all PCs. This figure provides a clear illustration on how the relative proportions are compared with the broken stick distributions and how the estimated number of PCs is chosen from the Broken-Stick model. Figure 2 illustrates how the Auer-Gervini model works. For the

simulated data set, there are $NS = 15$ objects, so the possible models $\mathcal{M}_d$ range from $\mathcal{M}_0$ to $\mathcal{M}_{14}$. The values $d = 0, 3, 4, 6, 7, 8, 11, 12, 13$ and 14 should be retained since the step functions are flat on those vertical coordinates. From the plot, we can see that the highest dimension d for which the step is significantly large is at $d = 0$. So $\mathcal{M}_0$ is a reasonable model.

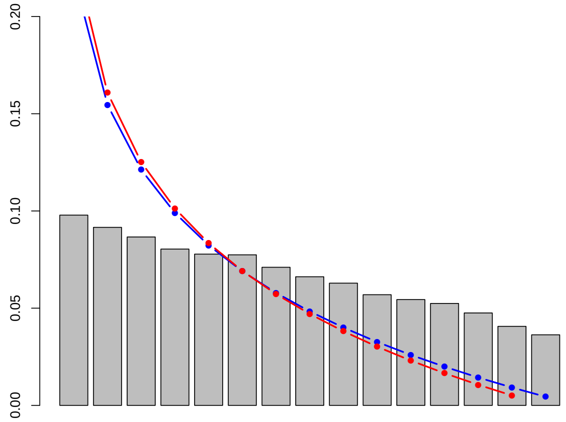

Supplementary Figure 1. Screeplot of the data and the Broken-Stick model.

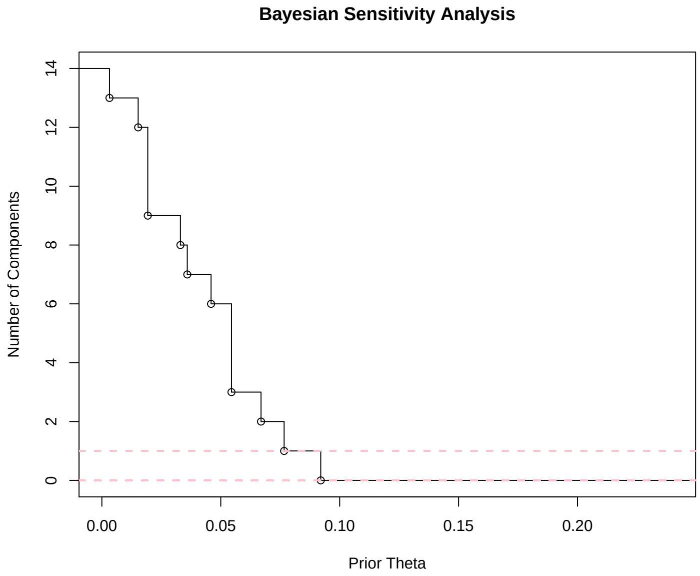

Supplementary Figure 2. Auer-Gervini step function relating the prior hyperparameter θ to the maximum posterior estimate of the number K of significant principal components.

S2. Tables for comparison of all types of methods without random noise (Tables 1 – 7)

Supplementary Table 1. Values of the absolute difference between the sample test estimates and the known dimension across the correlation matrices with data sets of different number of blocks (1X, 2X) and size (24×100), (96×100), (24×400) and (96×400). Bold values indicate the best results for column settings.

Rules	Subrules	Original Blocks (1X)				Twice Blocks (2X)			
		24 objects		96 objects		24 objects		96 objects	
		m=100	m=400	m=100	m=400	m=100	m=400	m=100	m=400
Bartlett	bartlett	5.67	6.57	49.94	23.72	5.34	5.65	47.96	23.37
	anderson	6.34	6.81	92.62	17.53	5.87	5.84	90.51	18.31
	lawley	3.57	4.49	60.6	11.31	4.01	4.56	58.52	12.1
Broken-Stick	broken.stick	0.67	0.78	**0.4**	0.57	2.49	2.79	**0.89**	**1.1**
Rand.-based	rnd.Lambda	0.67	0.63	0.62	**0.55**	1.59	1.44	1.33	1.21
	rnd.F	3.8	4.44	10.83	15.44	4.53	5.29	12.32	18.01
Minka	laplace	1.12	1.51	0.59	1.57	**0.88**	1.12	1.13	1.17
GCV	gcv	0.72	0.53	1.51	1.48	1.34	**1.08**	2	1.81
PESEL	pesel.m.hete	0.63	0.94	0.68	0.63	1.93	1.13	1.63	1.27
	pesel.n.hete	0.94	0.86	0.85	0.84	2.38	2.02	1.88	1.65
	pesel.n.homo	0.94	0.86	0.85	0.84	2.38	2.03	1.89	1.65
Auer-Gervini	twicemean	**0.44**	0.46	6.21	1.44	1.48	1.28	7.36	1.99
	spectral	0.69	0.54	7.04	2.66	2.71	2.47	8.86	3.29
	kmeans	0.54	0.58	3.25	0.63	1.71	1.63	3.91	1.4
	kmeans3	0.54	0.58	3.25	3.18	1.71	1.63	3.91	3.82
	ttest	6.89	7.6	68.12	24.75	5.93	5.99	59.86	23.2
	ttest2	0.51	**0.4**	5.08	0.73	2.37	2.04	7.95	3.1
	cpt	0.63	0.68	0.94	0.61	1.65	1.5	1.72	1.35
	cpmfun	2.9	3.26	47.7	15.65	4.31	3.22	51.53	15.65
	majority	0.51	0.5	5.36	1.07	2.21	2.05	7.02	1.77

Supplementary Table 2. Percentage of deviations between the estimate from randomization-based procedure (rnd-Lambda) and known dimension.

Matrix	24 objects (m=100)							24 objects (m=400)						
	≤ -3	-2	-1	0	1	2	≥ 3	≤ -3	-2	-1	0	1	2	≥ 3
1	52	41.5	6.5	0	0	0	0	2.4	84	13.6	0	0	0	0
2	100	0	0	0	0	0	0	100	0	0	0	0	0	0
3	100	0	0	0	0	0	0	100	0	0	0	0	0	0
4	0	0	0	94.6	4.7	0.5	0.2	0	0	0	95.4	4.6	0	0
5	0	0	0	100	0	0	0	0	0	0	100	0	0	0
6	0	0	0	100	0	0	0	0	0	0	100	0	0	0
7	0	0	0	100	0	0	0	0	0	0	100	0	0	0
8	0	0	0	100	0	0	0	0	0	0	100	0	0	0
9	0	0	0	100	0	0	0	0	0	0	100	0	0	0
10	0	0	0.1	97.1	2.8	0	0	0	0	0	99	1	0	0
11	0	0	0.1	96.1	3.8	0	0	0	0	0	98.2	1.8	0	0
12	0	0	0	93.4	5.8	0.8	0	0	0	0	95	4.6	0.2	0.2
13	0	0	0	85.8	12.6	1.5	0.1	0	0	0	90.6	8	1.4	0
14	0	0	0	100	0	0	0	0	0	0	100	0	0	0
15	0	0	0	100	0	0	0	0	0	0	100	0	0	0
16	0	0	0	100	0	0	0	0	0	0	100	0	0	0
17	0	0	0	100	0	0	0	0	0	0	100	0	0	0
18	0	0	0	100	0	0	0	0	0	0	100	0	0	0
19	0	0	0	100	0	0	0	0	0	0	100	0	0	0

Supplementary Table 3. Percentage of deviations between the estimate from randomization-based procedure (rnd-Lambda) and known dimension.

Matrix	96 objects (m=100)							96 objects (m=400)						
	≤ -3	-2	-1	0	1	2	≥ 3	≤ -3	-2	-1	0	1	2	≥ 3
1	11.1	58.4	27.5	2.9	0.1	0	0	0	0.2	53.6	46.2	0	0	0
2	100	0	0	0	0	0	0	100	0	0	0	0	0	0
3	100	0	0	0	0	0	0	100	0	0	0	0	0	0
4	0	0	0	94.7	4.4	0.5	0.4	0	0	0	93.6	5.4	0.8	0.2
5	0	0	0	100	0	0	0	0	0	0	100	0	0	0
6	0	0	0	100	0	0	0	0	0	0	100	0	0	0
7	0	0	0	100	0	0	0	0	0	0	100	0	0	0
8	0	0	0	100	0	0	0	0	0	0	100	0	0	0
9	0	0	0	100	0	0	0	0	0	0	100	0	0	0
10	0	0	0	100	0	0	0	0	0	0	100	0	0	0
11	0	0	0	100	0	0	0	0	0	0	100	0	0	0
12	0	0	0	99.8	0.2	0	0	0	0	0	100	0	0	0
13	0	0	0	96.6	3.1	0.3	0	0	0	0	99	1	0	0
14	0	0	0	100	0	0	0	0	0	0	100	0	0	0
15	0	0	0	100	0	0	0	0	0	0	100	0	0	0
16	0	0	0	100	0	0	0	0	0	0	100	0	0	0
17	0	0	0	100	0	0	0	0	0	0	100	0	0	0
18	0	0	0	100	0	0	0	0	0	0	100	0	0	0
19	0	0	0	100	0	0	0	0	0	0	100	0	0	0

Supplementary Table 4. Percentage of deviations between the estimate from the “CPT” criterion in the Auer-Gervini model and known dimension.

Matrix	24 objects (m=100)							24 objects (m=400)						
	≤ -3	-2	-1	0	1	2	≥ 3	≤ -3	-2	-1	0	1	2	≥ 3
1	94.8	4.5	0.7	0	0	0	0	99.6	0.4	0	0	0	0	0
2	91.1	7.7	1.1	0	0	0	0	97.8	1.6	0.6	0	0	0	0
3	82.8	13.1	3.1	0.9	0	0.1	0	86.8	10.6	2.2	0.4	0	0	0
4	0	0	0	94.2	5.1	0.5	0.2	0	0	0	94.2	7.2	0.4	0
5	0	0	0	100	0	0	0	0	0	0	100	0	0	0
6	0	0	0	100	0	0	0	0	0	0	100	0	0	0
7	0	0	0	100	0	0	0	0	0	0	100	0	0	0
8	0	1.8	4.2	94	0	0	0	0	0	0	100	0	0	0
9	0	0	0	100	0	0	0	0	0	0	100	0	0	0
10	0	0	50.2	49.8	0	0	0	0	0	36.3	63.7	0	0	0
11	0	0	35.6	64.4	0	0	0	0	0	10.3	89.7	0	0	0
12	0	0	0	100	0	0	0	0	0	0	100	0	0	0
13	0	0	0	99.9	0.1	0	0	0	0	0	100	0	0	0
14	0	0	0	100	0	0	0	0	0	0	100	0	0	0
15	0	0	0	100	0	0	0	0	0	0	100	0	0	0
16	0	0	0	100	0	0	0	0	0	0	100	0	0	0
17	0	0	0.1	99.9	0	0	0	0	0	0	100	0	0	0
18	0	0	0	100	0	0	0	0	0	0	100	0	0	0
19	0	0	0.1	99.9	0	0	0	0	0	0	100	0	0	0

Supplementary Table 5. Percentage of deviations between the estimate from the "CPT" criterion in the Auer-Gervini model and known dimension.

Matrix	96 objects (m=100)							96 objects (m=400)						
	≤ -3	-2	-1	0	1	2	≥ 3	≤ -3	-2	-1	0	1	2	≥ 3
1	97	2.7	0	0	0	0	0.3	99.8	0.2	0	0	0	0	0
2	93.5	6.0	0.2	0	0	0	0.3	97.4	2.4	0.3	0	0	0	0
3	82	11.3	3.7	1.3	0.7	0.1	0.8	82.8	10.8	5	1.2	0.2	0	0
4	0	0	0	89.8	1.5	0.1	8.5	0	0	0	90.8	8	0.8	0.4
5	0	0	0	100	0	0	0	0	0	0	100	0	0	0
6	0	0	0	100	0	0	0	0	0	0	100	0	0	0
7	0	0	0	100	0	0	0	0	0	0	100	0	0	0
8	0	0	0	100	0	0	0	0	0	0	100	0	0	0
9	0	0	0	100	0	0	0	0	0	0	100	0	0	0
10	0	0	19.1	80.9	0	0	0	0	0	1.2	98.8	0	0	0
11	0	0	10.5	89.5	0	0	0	0	0	0	100	0	0	0
12	0	0	0	100	0	0	0	0	0	0	100	0	0	0
13	0	0	0	100	0	0	0	0	0	0	100	0	0	0
14	0	0	0	100	0	0	0	0	0	0	100	0	0	0
15	0	0	0	100	0	0	0	0	0	0	100	0	0	0
16	0	0	0	100	0	0	0	0	0	0	100	0	0	0
17	0	0	0	100	0	0	0	0	0	0	100	0	0	0
18	0	0	0	100	0	0	0	0	0	0	100	0	0	0
19	0	0	0	100	0	0	0	0	0	0	100	0	0	0

Supplementary Table 6. Percentage of deviations between the estimate from "TwiceMean" criterion in the Auer-Gervin model and known dimension.

Matrix	24 objects (m=100)							24 objects (m=400)						
	≤ -3	-2	-1	0	1	2	≥ 3	≤ -3	-2	-1	0	1	2	≥ 3
1	58	28.3	12	1.7	0	0	0	65.4	27.8	3.4	3.4	0	0	0
2	51.7	30.1	13.6	4.6	0	0	0	53.3	28.7	11.3	6.7	0	0	0
3	52.5	24.8	12.5	6.8	2.6	0.6	0.2	47.1	29.3	13.8	7.2	2.2	0.4	0
4	0	0	0	51.7	21.8	11.2	15.3	0	0	0	36.1	27.5	12.2	24.2
5	0	0	0	100	0	0	0	0	0	0	100	0	0	0
6	0	0	0	100	0	0	0	0	0	0	100	0	0	0
7	0	0	0	100	0	0	0	0	0	0	100	0	0	0
8	0	0	0	98.8	1.2	0	0	0	0	0	100	0	0	0
9	0	0	0	100	0	0	0	0	0	0	100	0	0	0
10	0	0	13	87	0	0	0	0	0	16.9	83.1	0	0	0
11	0	0	7.3	92.7	0	0	0	0	0	5.1	94.9	0	0	0
12	0	0	0	100	0	0	0	0	0	0	100	0	0	0
13	0	0	0	88	8.8	2.2	1	0	0	0	100	0	0	0
14	0	0	0	100	0	0	0	0	0	0	100	0	0	0
15	0	0	0	100	0	0	0	0	0	0	100	0	0	0
16	0	0	0	100	0	0	0	0	0	0	100	0	0	0
17	0	0	0	100	0	0	0	0	0	0	100	0	0	0
18	0	0	0	100	0	0	0	0	0	0	100	0	0	0
19	0	0	0	100	0	0	0	0	0	0	100	0	0	0

Supplementary Table 7. Percentage of deviations between the estimate from the Broken-Stick model and known dimension.

Matrix	96 objects (m=100)							96 objects (m=400)						
	≤ -3	-2	-1	0	1	2	≥ 3	≤ -3	-2	-1	0	1	2	≥ 3
1	80.1	19.5	0.4	0	0	0	0	100	0	0	0	0	0	0
2	72.1	25.4	2.5	0	0	0	0	100	0	0	0	0	0	0
3	29.5	25.1	20.4	12.9	7.4	3.3	1.4	85.4	11.2	2.8	0.6	0	0	0
4	0	0	0	100	0	0	0	0	0	0	100	0	0	0
5	0	0	0	100	0	0	0	0	0	0	100	0	0	0
6	0	0	0	100	0	0	0	0	0	0	100	0	0	0
7	0	0	0	100	0	0	0	0	0	0	100	0	0	0
8	0	0	0	100	0	0	0	0	0	0	100	0	0	0
9	0	0	0	100	0	0	0	0	0	0	100	0	0	0
10	0	0	0	100	0	0	0	0	0	0	100	0	0	0
11	0	0	0	100	0	0	0	0	0	0	100	0	0	0
12	0	0	0	100	0	0	0	0	0	0	100	0	0	0
13	0	0	0	100	0	0	0	0	0	0	100	0	0	0
14	0	0	0	100	0	0	0	0	0	0	100	0	0	0
15	0	0	0	100	0	0	0	0	0	0	100	0	0	0
16	0	0	0	100	0	0	0	0	0	0	100	0	0	0
17	0	0	0	100	0	0	0	0	0	0	100	0	0	0
18	0	0	0	100	0	0	0	0	0	0	100	0	0	0
19	0	0	0	100	0	0	0	0	0	0	100	0	0	0

S3. Tables for comparing all types of methods under different levels of noise (Tables 8 – 10)

Supplementary Table 8. Values of the absolute difference between the sample test estimates and the known dimension across the correlation matrices with data sets of different scenarios under noise level $\sigma_e^2 = 0.01$. Bold values indicate the best results for column settings.

Rules	Subrules	Original Blocks (1X)				Twice Blocks (2X)			
		24 objects		96 objects		24 objects		96 objects	
		m=100	m=400	m=100	m=400	m=100	m=400	m=100	m=400
Bartlett	bartlett	5.56	6.2	49.52	23.72	5.33	5.79	47.96	23.36
	anderson	6.09	6.47	92.61	17.32	5.88	5.98	90.51	18.3
	lawley	3.36	4.14	60.21	11.23	4	4.68	58.57	11.97
Broken-Stick	broken.stick	0.69	0.8	**0.43**	0.59	2.54	2.85	**0.95**	**1.17**
Rand.-based	rnd.Lambda	0.66	0.62	0.62	**0.55**	1.6	1.47	1.35	1.22
	rnd.F	3.81	4.41	10.63	15.5	4.54	5.27	12.21	17.96
Minka	laplace	1.07	1.49	0.61	1.55	**1**	**1.12**	1.23	1.25
GCV	gcv	0.69	0.52	1.48	1.43	1.44	1.2	2.02	1.82
PESEL	pesel.m.hete	0.63	0.79	0.68	0.63	2.02	1.13	1.66	1.31
	pesel.n.hete	0.97	0.87	0.85	0.84	2.44	2.23	1.91	1.68
	pesel.n.homo	0.96	0.87	0.85	0.84	2.43	2.22	1.92	1.68
Auer-Gervini	twicemean	**0.44**	0.44	6.65	1.55	1.47	1.31	8.03	2.08
	spectral	0.69	0.56	7.67	2.64	2.68	2.54	9.39	3.25
	kmeans	0.54	0.56	3.37	0.64	1.7	1.65	3.84	1.41
	kmeans3	0.54	0.56	3.37	3.25	1.7	1.65	3.84	4
	ttest	6.64	7.34	68.64	24.6	5.9	5.93	60.69	23.51
	ttest2	0.49	**0.38**	5.16	0.68	2.3	2.04	7.94	3.04
	cpt	0.62	0.67	0.98	0.62	1.67	1.5	1.68	1.38
	cpmfun	2.67	3.17	48.52	15.54	4.32	3.27	53.61	15.56
	majority	0.52	0.5	5.71	1.12	2.14	2.05	7.23	1.81

Supplementary Table 9. Values of the absolute difference between the sample test estimates and the known dimension across the correlation matrices with data sets of different scenarios under noise level $\sigma_e^2 = 0.1$. Bold values indicate the best results for column settings.

Rules	Subrules	Original Blocks (1X)				Twice Blocks (2X)			
		24 objects		96 objects		24 objects		96 objects	
		m=100	m=400	m=100	m=400	m=100	m=400	m=100	m=400
Bartlett	bartlett	5.47	5.98	49.26	23.6	5.46	5.33	47.55	23.19
	anderson	5.97	6.22	92.61	16.7	5.77	5.53	90.51	16.93
	lawley	3.23	3.89	60.15	10.95	3.93	4.23	58.4	11.47
Broken-Stick	broken.stick	0.89	0.95	**0.65**	0.75	2.85	3.26	**1.35**	1.51
Rand.-based	rnd.Lambda	0.68	0.63	**0.65**	**0.59**	1.66	1.47	1.4	**1.3**
	rnd.F	3.78	4.27	9.39	14.96	4.63	5.19	11.04	17.43
Minka	laplace	0.77	1.47	0.68	1.64	1.46	1.5	1.53	1.9
GCV	gcv	0.69	0.62	1.28	1.03	1.66	1.5	1.97	1.68
PESEL	pesel.m.hete	0.7	0.61	0.68	0.68	2.24	1.39	1.81	1.44
	pesel.n.hete	1.02	0.97	0.86	0.84	2.56	2.49	2.05	1.78
	pesel.n.homo	1.02	0.97	0.87	0.84	2.56	2.49	2.05	1.78
Auer-Gervini	twicemean	**0.48**	**0.49**	10.92	1.53	**1.39**	**1.36**	12.63	2.17
	spectral	0.75	0.7	11.57	2.72	2.23	2.46	13.98	3.48
	kmeans	0.55	0.59	4.86	0.64	1.66	1.65	4.83	1.45
	kmeans3	0.55	0.59	4.86	3.21	1.66	1.65	4.83	3.98
	ttest	5.88	7.32	68.94	21.12	5.48	6.12	63.11	20.48
	ttest2	0.52	0.51	6.98	0.61	1.87	2.07	10.36	1.42
	cpt	0.63	0.67	1.27	0.62	1.8	1.68	1.93	1.42
	cpmfun	2.12	3.22	51.07	10.03	4.28	3.63	56.57	11.35
	majority	0.56	0.56	9.21	1.18	1.84	1.93	10.51	1.92

Supplementary Table 10. Values of the absolute difference between the sample test estimates and the known dimension across the correlation matrices with data sets of different scenarios under noise level $\sigma_e^2 = 1$. Bold values indicate the best results for column settings.

Rules	Subrules	Original Blocks (1X)				Twice Blocks (2X)			
		24 objects		96 objects		24 objects		96 objects	
		m=100	m=400	m=100	m=400	m=100	m=400	m=100	m=400
Bartlett	bartlett	4.84	5.09	48.04	18.87	5.21	5.05	46.06	17.98
	anderson	5.14	5.4	92.61	10.5	5.19	5.29	90.51	10.86
	lawley	1.41	2.87	59.35	3.59	2.56	3.7	57.21	4.34
Broken-Stick	broken.stick	1.42	1.48	0.79	0.8	3.81	4.05	2.02	2.6
Rand.-based	rnd.Lambda	0.83	0.71	0.79	0.72	2.29	1.6	1.66	1.51
	rnd.F	1.73	3.86	2.64	10.48	2.97	4.99	4.95	13.17
Minka	laplace	0.76	0.9	**0.7**	0.69	2.34	1.7	2.2	1.48
GCV	gcv	0.76	0.7	0.85	**0.68**	2.39	2.31	**1.65**	**1.47**
PESEL	pesel.m.hete	0.98	0.69	0.82	**0.68**	2.96	2.27	2.43	1.68
	pesel.n.hete	1.12	1.1	1.04	0.98	3.11	2.91	2.64	2.42
	pesel.n.homo	1.12	1.1	1.04	0.98	3.11	2.91	2.63	2.42
Auer-Gervini	twicemean	**0.67**	**0.54**	17.58	3.99	**1.91**	**1.56**	25.37	3.43
	spectral	1.43	0.81	17.89	7.26	2.73	1.96	26.37	7.27
	kmeans	0.77	0.66	10.19	0.76	2.09	1.78	15.81	1.7
	kmeans3	0.77	0.66	10.19	10.96	2.09	1.78	15.81	10.29
	ttest	5.37	5.87	66.31	16.41	4.96	5.07	58.9	15.68
	ttest2	0.96	0.63	13.14	1.26	2.44	1.82	21.17	1.73
	cpt	0.91	0.8	2.66	**0.68**	2.32	1.93	4.53	1.9
	cpmfun	3.91	2.04	59.95	17.53	5.46	3.37	62.77	19.05
	majority	0.84	0.6	15.84	2.52	2.41	1.86	24.96	2.2

S4. Complete results for RPPA apoptosis analysis

Supplementary Table 11. Number of principal components (PCs) from different algorithms on RPPA data

Rules	Auer-Gervini							
	twicemean	spectral	kmeans	kmeans3	ttest	ttest2	cpt	cpmfun
PCs	6	6	1	1	4	1	1	10
Rules	Bartlett			Broken-Stick	Rand.-based		Minka	GCV
	bartlett	anderson	lawley	broken-stick	rnd-Lambda	rnd-F	laplace	gcv
PCs	30	30	30	1	8	31	20	12
Rules	PESEL							
	pesel.m.hete	pesel.n.hete	pesel.n.homo					
PCs	15	1	1					

S5. Histogram plot of all methods in a special scenario

That is, histogram of the estimated dimension for 19 covariance and correlation structures from all types of methods over 1000 samples (n=24, m=100) is provided in Supplementary Figure 3.

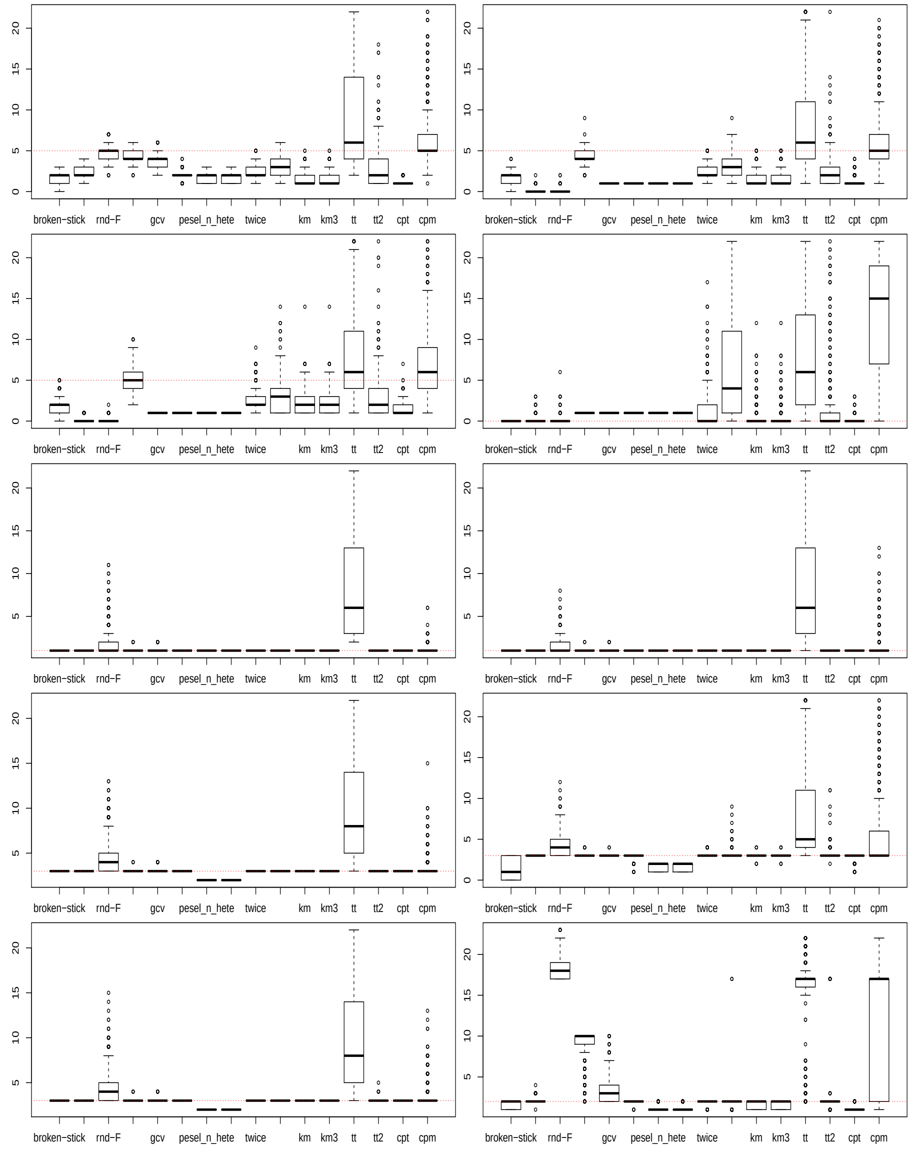

Supplementary Figure 3. Histogram of the estimated dimension for 19 covariance and correlation structures from different methods. Within each plot, from left to right, we show (1) Broken-Stick, (2–3) randomization based procedures, (4) Minka's Laplace approximation, (5) generalized cross-validation, (6–8) PESEL criteria, and (9–16) the Auer-Gervini model with different criteria. Horizontal dotted lines correspond to the true number of PCs over 1000 samples (n=24, m=100).

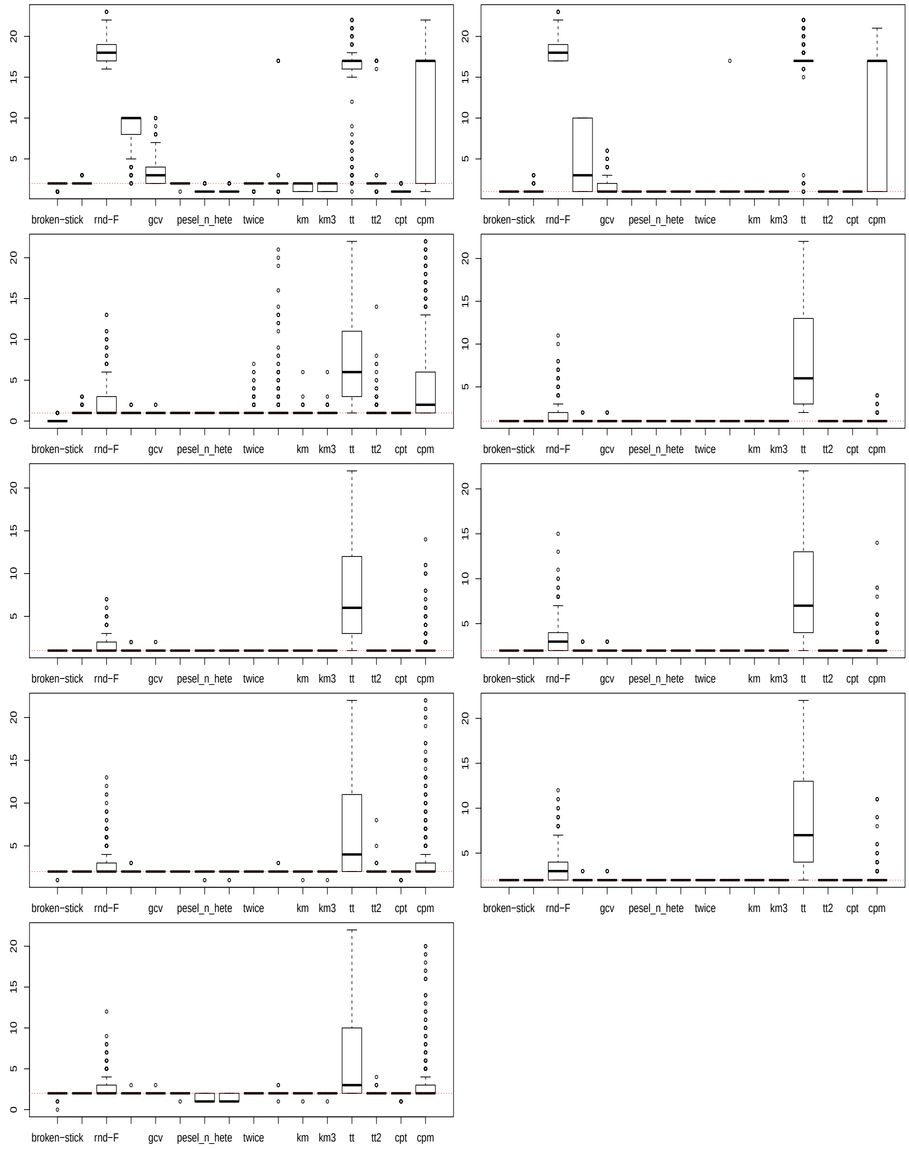

Supplementary Figure 3. (Continued) Histogram of the estimated dimension for 19 covariance and correlation structures from different methods. Within each plot, from left to right, we show (1) Broken-Stick, (2–3) randomization based procedures, (4) Minka's Laplace approximation, (5) generalized cross-validation, (6–8) PESEL criteria, and (9–16) the Auer-Gervini model with different criteria. Horizontal dotted lines correspond to the true number of PCs over 1000 samples (n=24, m=100).
